# The environmental carcinogen benzo[a]pyrene induces a Warburg-like metabolic reprogramming dependent on NHE1 and associated with cell survival

**DOI:** 10.1038/srep30776

**Published:** 2016-08-04

**Authors:** Kévin Hardonnière, Elise Saunier, Anthony Lemarié, Morgane Fernier, Isabelle Gallais, Cécile Héliès-Toussaint, Baharia Mograbi, Samantha Antonio, Paule Bénit, Pierre Rustin, Maxime Janin, Florence Habarou, Chris Ottolenghi, Marie-Thérèse Lavault, Chantal Benelli, Odile Sergent, Laurence Huc, Sylvie Bortoli, Dominique Lagadic-Gossmann

**Affiliations:** 1 Institut national de la santé et de la recherche médicale (Inserm), Institut de recherche en santé, environnement et travail (Irset – Inserm UMR 1085), F-35043 Rennes, France; 2 Université de Rennes 1, Structure fédérative de recherche Biosit, UMS CNRS 3480/US Inserm 018, F 35043 Rennes, France; 3 Inserm UMR 1124, Université Paris Descartes, Centre Universitaire des Saints-Pères, Paris, France; 4 Inserm-U1037, University of Toulouse, Cancer Research Center of Toulouse CRCT, Department of Experimental Therapeutics 2 av. Hubert Curien, 31100 Toulouse, France; 5 INRA UMR 1331 ToxAlim (Research Center in Food Toxicology), University of Toulouse ENVT, INP, UPS, 180 Chemin de Tournefeuille, F-31027, France; 6 Institute of Research on Cancer and Ageing of Nice (IRCAN), INSERM U1081, CNRS UMR7284, Université de Nice-Sophia Antipolis, Faculté de Médecine, Centre Antoine Lacassagne, Nice, F-06107, France; 7 Hôpital Robert Debré, INSERM UMR1141, Bâtiment Ecran, 48 Boulevard Sérurier, Paris 75019, France; 8 U.F.R. de Médecine Université Paris Diderot, Paris 75019, France; 9 Biochimie Métabolique, Assistance Publique-Hôpitaux de Paris, Groupe Hospitalier Necker-Enfants Malades, Paris, France

## Abstract

Cancer cells display alterations in many cellular processes. One core hallmark of cancer is the Warburg effect which is a glycolytic reprogramming that allows cells to survive and proliferate. Although the contributions of environmental contaminants to cancer development are widely accepted, the underlying mechanisms have to be clarified. Benzo[a]pyrene (B[a]P), the prototype of polycyclic aromatic hydrocarbons, exhibits genotoxic and carcinogenic effects, and it is a human carcinogen according to the International Agency for Research on Cancer. In addition to triggering apoptotic signals, B[a]P may induce survival signals, both of which are likely to be involved in cancer promotion. We previously suggested that B[a]P-induced mitochondrial dysfunctions, especially membrane hyperpolarization, might trigger cell survival signaling in rat hepatic epithelial F258 cells. Here, we further characterized these dysfunctions by focusing on energy metabolism. We found that B[a]P promoted a metabolic reprogramming. Cell respiration decreased and lactate production increased. These changes were associated with alterations in the tricarboxylic acid cycle which likely involve a dysfunction of the mitochondrial complex II. The glycolytic shift relied on activation of the Na^+^/H^+^ exchanger 1 (NHE1) and appeared to be a key feature in B[a]P-induced cell survival related to changes in cell phenotype (epithelial-to-mesenchymal transition and cell migration).

Metabolic reprogramming upon malignant transformation has been extensively studied. The reversible metabolic shift from oxidative phosphorylation (OXPHOS) to aerobic glycolysis (Warburg effect) is now a core hallmark of cancer cells^1^ that supports survival and neoplastic proliferation^2^.

More recently, close interconnections between energy metabolism and cell fate have been reported in which mitochondria play a crucial role, notably through a number of death effectors and the control of organic acid balances^3^,^4^. In line with this, a high mitochondrial membrane potential (Δψm) appears to be a relevant marker for mitochondrial dysfunction in cancer. Indeed, many carcinomas display high Δψm^5^, and cells with high Δψm appear to be more prone to form tumors^6^,^7^. Interestingly, a high Δψm has been measured concomitantly to cell metabolic reprogramming towards glycolysis in human hepatocarcinoma HepG2 cells^8^.

Environmental carcinogens are among the various factors which might favor a high Δψm and hence metabolic reprogramming. Δψm increased following activation of the aryl hydrocarbon receptor (AhR) by 2,3,7,8 tetrachlorodibenzo-p-dioxin (TCDD) in murine hepatoma Hepa1c1c7 cells^9^. However, it remains to be determined whether glycolytic reprogramming occurs following activation of the AhR. AhR is activated by several polycyclic aromatic hydrocarbons (PAHs) which are major environmental contaminants that are found in exhaust fume, cigarette smoke and diet. The PAH prototype benzo[a]pyrene (B[a]P), an AhR ligand, exhibits a strong carcinogenic potential, and it is classified as a carcinogen to humans by the International Agency for Research on Cancer (IARC). However, B[a]P carcinogenicity implies diverse mechanisms which are not fully understood. Following its bioactivation *via* cytochromes P450, B[a]P is genotoxic, and, hence, could lead to gene mutations, eg. in *p53*^10^. It also could favor cancer promotion by conferring a selective advantage to the initiated cells, notably through the expression of growth factors and/or anti-apoptotic proteins. The death of normal cells upon exposure to B[a]P might contribute in addition to the proliferation of pre-neoplastic cells^11^. B[a]P also can favor metastasis *via* the promotion of cell migration^12^ or by acting on the expression of extracellular matrix components^13^. Regarding cell metabolism, we have shown previously, in rat epithelial hepatic F258 cells, that B[a]P can affect lipid metabolism^14^, and the expression of hexokinase II, c-myc and GSK3 proteins^15^,^16^, all of which are known to control energy metabolism^17^,^18^. Further, activation of the Na^+^/H^+^ exchanger 1 (NHE1) by B[a]P leads to intracellular alkalinization^15^, an event known to play a role in metabolic reprogramming and malignant transformation^19^. However, the effects of B[a]P, and of PAHs more generally, on cell energy metabolism are not well known. Since exposure to B[a]P leads to mitochondrial hyperpolarization in F258 cells^20^, possibly in concert with activation of a survival pathway^21^, we hypothesized that a glycolytic shift might occur upon exposure to B[a]P.

Considering the B[a]P-induced hyperpolarization of F258 cells, we here investigated the effects of this carcinogen on energy metabolism of these cells. F258 cells also are sensitive to low concentrations of B[a]P, more relevant to environmental exposure^22^. Our study revealed that B[a]P induced a metabolic reprogramming that involved the activation of NHE1^15^,^23^, and that it led to the appearance of an epithelial-mesenchymal transition (EMT) phenotype.

## Methods

### Chemicals

Benzo[a]pyrene (B[a]P), 7,12-Dimethylbenz[a]anthracene (DMBA), α-naphthoflavone (α-NF), cytochalasin B, insulin, 2-deoxyglucose and 2,3,7,8-Tetrachlorodibenzo-p-dioxin (TCDD) were purchased from Sigma-Aldrich (Saint Quentin Fallavier, France). N-(Diaminomethylene)-4-isopropyl-3-(methylsulfonyl)benzamide (Cariporide) was purchased from Santa Cruz Biotechnology (Heidelberg, Germany). Hoechst 33342 was purchased from Life Technologies (Les Ulis, France). All these products were used as a stock solution in DMSO; the final concentration of this vehicle in the culture medium was <0.00005% (v/v), and control cultures received the same concentration of vehicle as treated cultures. [^3^H]-2-deoxyglucose was from PerkinElmer (Boston and Waltham, USA). Monoclonal mouse anti-HSC70 antibody (sc-7298) and monoclonal mouse anti-actin antibody (sc-8432) were purchased from Santa Cruz Biotechnology. Monoclonal mouse anti-E-Cadherin antibody (610404) was purchased from BD Biosciences (Le Pont de Claix, France), and monoclonal mouse anti-vimentin antibody (M0725) and secondary antibodies conjugated with horseradish peroxidase, from DAKO (Les Ulis, France).

### Cell culture

The F258 rat liver epithelial cell line (cf.^21^, for further details) was cultured in Williams’ E medium supplemented with 10% fetal calf serum (FCS) and 2 mM L-glutamine. When necessary, F258 cells were grown in galactose media: Williams’E medium deprived of glucose, supplemented with 11 mM galactose, 10% fetal calf serum and 2 mM glutamine. For this condition, F258 cells were grown in galactose medium for at least three passages before the experiments were conducted. The mouse hepatoma Hepa1c1c7 cells were grown in alpha MEM medium supplemented with 10% FCS and 2 mM L-glutamine. Each cell line was grown at 37 °C under a 5% CO_2_ atmosphere, and treated 24 h following seeding with B[a]P for 24, 48 and 72 h, as previously described^23^.

### Analysis of oxygen consumption and extracellular acidification rates

F258 cells were seeded in Seahorse XF 24-well microplates (Proteigene, St Marcel, France) at 6000 cells/well. Twenty four hours later, cells were treated with 50 nM B[a]P or DMSO for 48 h. On the day prior to the experiment, XF extracellular flux cartridge was hydrated with XF calibrant overnight. After a 48 h-treatment, the medium was changed to assay medium (unbuffered DMEM with 10 mM glucose, 2 mM glutamine, 2 mM pyruvate) and kept 1 h in a non-CO_2_ incubator at 37 °C. The mitochondrial function test was performed with consecutive injections of inhibitors of the electron transport chain (ETC): 1 μM oligomycin (inhibitor of the ATP synthase), 0.5 μM FCCP (Carbonyl cyanide 4-(trifluoromethoxy)phenylhydrazone) (uncoupler of the mitochondrial inner membrane, to get the maximum electron flux through ETC), and a mix of 1 μM rotenone/1μM antimycin A (inhibition of complex I and III of ETC). OCR (oxygen consumption rate) and ECAR (extracellular acidification rate) were measured and normalized according to protein content. The individual parameters (basal OCR, maximal OCR and reserve capacity) were determined by calculating the area under the curves, as previously described^24^. Five independent experiments were performed.

### Extracellular Lactate Measurement

Cell Supernatants were collected and directly frozen after culture experiments. L-lactate levels were measured using two enzymatic reactions. Lactate dehydrogenase (LDH; Roche, Meylan, France) catalyzed the NAD^+^ -mediated oxidation of lactate into pyruvate. Then glutamate-pyruvate transaminase (GPT; Roche, Meylan, France) was used to shift first reaction equilibrium by transforming all the pyruvate into alanine and α-ketoglutarate. The amount of NADH formed was related to the amount of lactate processed by these two coupled reactions. Briefly, 20 μL of each sample were added to 200 μL of reaction buffer (620 mM sodium carbonate, 78.7 mM L-glutamate, 0.92 mM NAD, 2 μg GPT and 2 μg LDH). A standard curve was obtained with lithium lactate (Sigma Aldrich). 96 multiwell plates were incubated at 37 °C for 30 minutes before quantifying extracellular lactate production by monitoring the increase in absorbance of NADH at 355 nm on a spectrophotometer (SpectraMax Gemini; Molecular Devices, France). At least three independent experiments, performed in triplicate and normalized to the protein content, were carried out for each experimental condition.

### Substrate oxidation assays

After a 48 h treatment with 50 nM B[a]P, 10^6^ isolated cells were incubated for 90 min at 37 °C in 1 mL of Krebs-Ringer phosphate buffer containing 5 mM U-^14^C-glucose (11 GBq/mmol, isotopic dilution 1/1000, Perkin Elmer), or 5 mM [U-^14^C] pyruvate (0.351 GBq/mmol, isotopic dilution 1/250, Perkin Elmer). CO_2_ was recovered for 1 h in benzethonium hydroxide after stopping the reaction with 6N sulphuric acid. The radioactive CO_2_ was counted by liquid scintillation (Ultima Gold, Perkin Elmer).

### Mitochondrial isolation and Complex II activity measurement

Mitochondria were isolated from B[a]P-treated (50 nM, 48 h) or untreated F258 cells, as previously described^25^, resuspended in lysis buffer and kept at −80 °C. The activities of complex II were measured spectrophotometrically^25^. Succinate dehydrogenase activity (SDH; subunits A-B) was determined by the reduction of DCPIP (dichlorophenolindophenol) in the presence of PMS (phenazine methosulfate) while succinate ubiquinone-reductase (SQR; subunits C-D) activity was assayed by the Coenzyme Q-dependent reduction of DCPIP. Briefly, for both activities, 10 μg of mitochondria were resuspended in 200 μL of phosphate buffer (35 mM, pH 7.3) supplemented with 2 mM KCN and 2 μg/mL Antimycin A (Sigma). For SDH activity, 10 mM succinate, 1.6 mM PMS and 40 μM DCPIP were added. For SQR activity, the mitochondrial suspension was combined with 40 mM succinate, 100 μM CoQ2 (Sigma C8081) and 88 μM DCPIP. Following a 5 min incubation at 37 °C, the absorbance at 610 nm was recorded every 30 s during 10 min (FLUOstar Optima, BMG Labtech), monitoring the extinction of DCPIP at 37 °C (ε = 21 mM^−1^ cm^−1^). Results were expressed as relative activities as compared to control cells. All reagents and chemicals were from Acros Organics (Fisher), unless stated.

### Measurement of succinate and fumarate concentrations

For organic acid analysis, 100 μl of cell pellet suspensions (2.9–4.4 μg/μl protein) were processed. To each sample, 2.4 nmoles of 4-phenylbutyric acid (P21005 from Sigma-Aldrich, France) and 1.5 nmoles of ^13^C_6_ labeled adipic acid (Eurisotop, Saint-Aubin, France) as internal standards were added. The organic fraction was extracted with ethylacetate, derivatized using N,Obis(trimethylsilyl) trifluoroacetamide with 1% trimethylchlorosilane, and analyzed by gas chromatography-tandem mass spectrometry on a Scion TQ triple quadrupole from Brüker Daltonics. Organic acids were identified according to retention time and at least two specific transitions (fumaric acid: 245 > 217 and 245 > 157; succinic acid: 247 > 131 and 172 > 112; labeled adipic acid 281 > 163 and 281 > 117; 4-phenylbutyric acid: 221 > 203 and 221 > 146).

### Transfection and RNA interference (siRNA)

ON-TARGETplus Rat AhR siRNA SMARTpool (siAhR) and ON-TARGETplus Non-Targeting Pool siRNA negative control (siNT) were purchased from GE Dharmacon. Basic small interfering RNA (siRNA) resuspension was realized according to manufacturer’s recommendations. Transfections with siRNA were performed in 60 mm dishes on 60% confluent F258 cells, in the presence of TransFectin Lipid Reagent (BioRad). Per dish, siRNA (100 nM) and 12.5 μl TransFectin lipid reagent were applied in a final volume of 2.5 ml Opti-MEM. Six hours later, the medium was renewed with the current medium as described above. Transfection rate was >86%. Cells were then passaged for treatment during the exponential phase.

### Cell toxicity estimation

Cell toxicity was measured by analysis of chromatin condensation and fragmentation in the cell nucleus, and ATP quantification, as previously described^21^.

### Real time cell impedance measurement

The xCELLigence system was used according to the manufacturer’s instructions (Roche Applied Science). Briefly, 2500 cells/well were seeded in 96-well E-plates in Williams’ E medium supplemented with 10% FCS, 2 mM L-glutamine. Twenty-four hours later, cells were treated with 50 nM B[a]P in the same medium. Cell impedance was measured in each well every 5 minutes for 80 hours. Impedance signals were analyzed by an integrated software (RTCA Analyzer), and expressed as a Cell Index (CI) value that reflects cell number, cell adhesion and/or cell morphology. The changes of cell impedance between control and treated cells were determined by calculating the slope of the line between two given time points.

### Flow cytometry analysis of the cell cycle

See Supplementary Information.

### Analysis of cell phenotype using Transmission Electron Microscopy (TEM)

See Supplementary Information.

### Analysis of cell migration

Cell migration was monitored using the xCELLigence system. Cells were serum starved 5 hours before seeding in a CIM-plate. Cells were trypsinized and treated with 500 nM B[a]P in Williams’ E medium without FCS, before seeding at 3.2 × 10^3^ cells/well in the upper chamber of a CIM-plate. Williams’ E medium supplemented with 10% fetal calf serum was added in the lower chamber of the CIM-plate. The impedance value of each well was measured every 5 minutes for 40 hours and expressed as a CI value.

### Western blotting

See Supplementary Information.

### Statistical analysis

All data were obtained from a minimum of three independent experiments. The mean ± standard deviation (SD) is shown in the figures. Analysis of variance followed by Newman–Keuls test was used to test the effects of B[a]P. Differences were considered significant at the level of *P* < 0.05. All statistical analyses were performed using GraphPad Prism 5.01 Software (GraphPad Software, San Diego, USA).

## Results

### Glycolytic shift induced by B[a]P

We first looked for evidence of glycolytic reprogramming using data from a previously published study of the effects of 2 μM B[a]P (72 h) on the transcriptome of human hepatocarcinoma HepG2 cells (GSE40117)^26^. More precisely, in order to identify the pathways that were differentially affected by exposure to B[a]P, we performed single-sample gene set enrichment analysis (ssGSEA) projection as a hypothesis-generating gene set identification tool. ssGSEA analysis revealed that the expression of an OXPHOS-related gene set was globally down-regulated, in contrast to glycolysis and xenobiotic metabolism-related genes which were up-regulated, thus indicating a B[a]P-induced metabolic reprogramming in hepatic cells (Supplementary Fig. S1).

**Figure 1:**
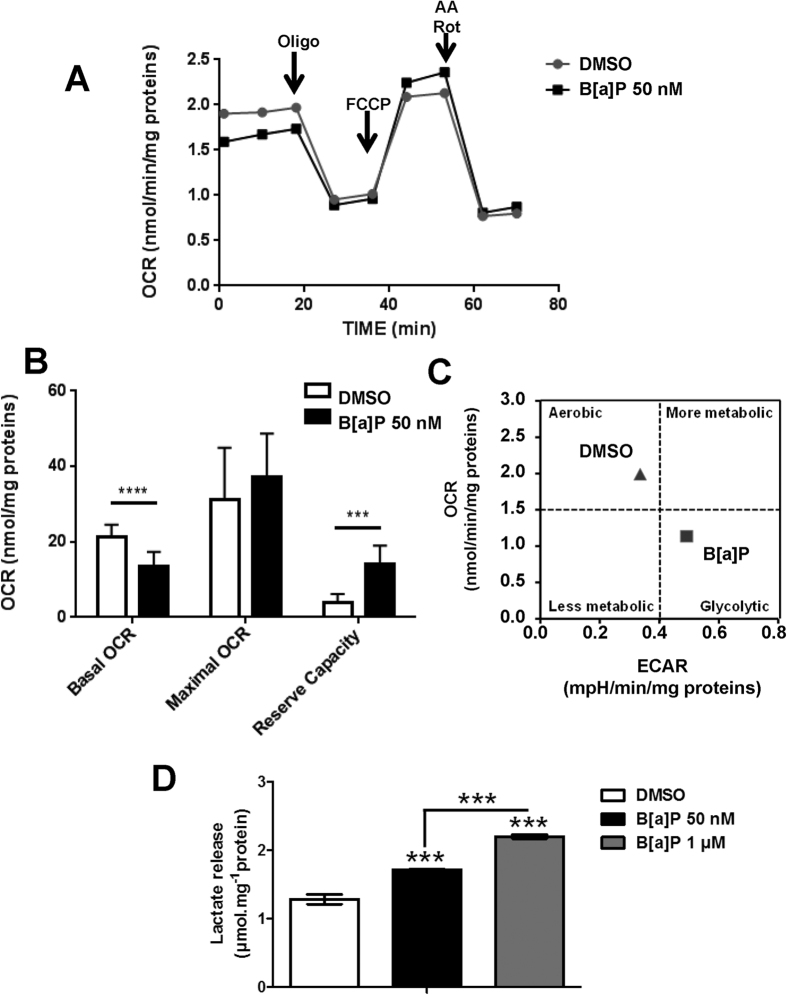
B[a]P induces a metabolic reprogramming towards glycolysis in F258 cells. (**A**) The impact of B[a]P (50 nM, 48 h) on the oxygen consumption rate (OCR; (**A,B**)) and the extracellular acidification rate (ECAR; C) was analyzed in F258 cells using XF Cell Mito Stress Test Kit and Seahorse XF24 technology. Oligo: oligomycin A; AA: antimycin A; Rot: rotenone. (**B**) Mitochondrial parameters (basal OCR, maximal OCR and Reserve Capacity) were calculated from the area under the curves as previously described^24^. (**C**) OCR values were plotted as a function of ECAR values in order to identify the metabolic profile of F258 cells exposed to B[a]P as compared to control (DMSO). (**D**) Extracellular lactate release was measured following 48 h-exposures to B[a]P (50 nM or 1 μM). N = 5 (**A–C**) and 4 (**D**) independent experiments. ***p < 0.001 and ****p < 0.0001: DMSO *vs* B[a]P-treated cells, unless indicated by lines on graph.

On the basis of these results, we then conducted a thorough characterization of the impact of a low concentration of B[a]P on energy metabolism in F258 cells. Oxygen consumption rate (OCR) analysis showed a strong inhibition of basal respiration following exposure to 50 nM B[a]P for 48 h (Fig. 1A,B). Although evaluation of the maximal respiration capacity with the uncoupler molecule FCCP showed a similar OCR between control and B[a]P-treated cells, the reserve capacity was significantly higher in B[a]P-treated cells (Fig. 1A,B). Therefore, B[a]P-treated cells would be better able to consume O_2_ upon increased ATP demand or during a stress, which suggests that these cells are better adapted to cope with other stress. Altogether, these data indicate that exposure to B[a]P leads to a change in energy metabolism.

We next measured the extracellular acidification rate (ECAR) and found that it was increased by B[a]P (Fig. 1C). Since extracellular acidification could reflect an accumulation of intracellular glycolytic by-products, we measured lactate release, a well-known indicator of glycolysis. Lactate release was enhanced by 50 nM B[a]P (48 h) with an even more pronounced increase with 1 μM (Fig. 1D). This dose-dependent effect was significant after 24 h of exposure and was enhanced after 72 h exposure (Supplementary Fig. S2A). We also found that B[a]P increased extracellular lactate in two other hepatic cell lines, HepG2 (Supplementary Fig. S2B) and Hepa1c1c7 (see below). Another carcinogenic PAH, DMBA (50 nM), exhibited effects on lactate production similar to B[a]P in F258 cells (Supplementary Fig. S2C).

Taken together, the results clearly show that exposure of F258 cells to B[a]P produces a glycolytic shift related to a major mitochondrial dysfunction.

### Alterations in the tricarboxylic acid cycle in F258 cells following exposure to B[a]P

**Figure 2:**
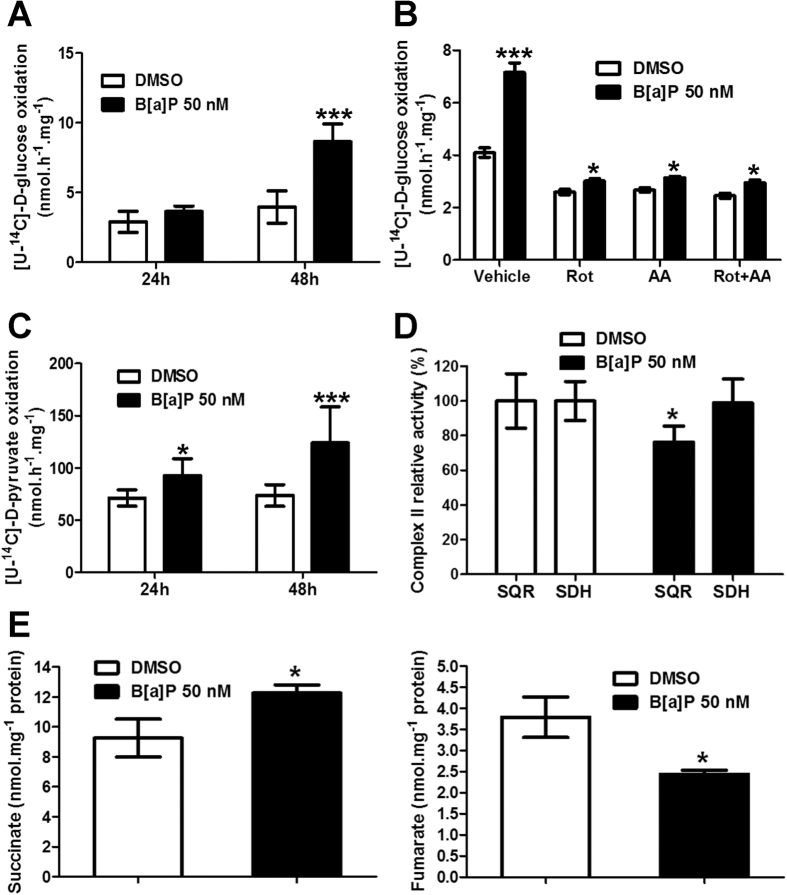
B[a]P is responsible for TCA cycle alterations by targeting mitochondrial complex II in F258 cells. (**A–C**) The effects of B[a]P (50 nM, 48 h) on substrate oxidation ((**A,B**) glucose; (**C**) pyruvate) were evaluated by quantifying the transfer of ^14^C from substrates to CO_2_. (**B**) Impact of OXPHOS inhibitors (ROT: rotenone, 1 μM; AA: Antimycin A, 1 μM) on the B[a]P (50 nM, 48 h)-induced increase in the level of glucose oxidation. N = 4 (**A,C**) and 3 (**B**) independent experiments. (**D**) Effects of B[a]P on the enzymatic activities of complex II. F258 cells were treated with B[a]P (50 nM, 48 h) and mitochondria were isolated. The SQR (succinate coenzyme Q oxidoreductase) and SDH (succinate dehydrogenase activity) activities of complex II were assessed by specific assays, as described in Methods. The data of five independent experiments, are given for Complex II relative activities as compared to the related control cells. *p < 0.05 compared with the related control. (**E**) Effects of B[a]P on fumarate and succinate levels were determined. N = 3 independent experiments. *p < 0.05; ***p < 0.001: DMSO *vs* B[a]P-treated cells for (**A–C,E**); for (**B**): inhibitor *vs* B[a]P+ inhibitor-treated cells.

In order to obtain further insight into the effects of B[a]P on glucose metabolism, we next assessed glucose oxidation by measuring the production of ^14^CO_2_. Oxidative capacity was significantly increased in F258 cells treated with 50 nM B[a]P for 48 h (Fig. 2A). We next determined whether the stimulation of both glucose oxidation and lactate production was linked to an increased glucose uptake. As expected, cytochalasin B, by inhibiting actin filament formation, blocked this uptake whereas B[a]P (50 nM, 48 h) was ineffective (Supplementary Fig. S3A). We then hypothesized that stimulation of the pentose phosphate pathway (PPP) might participate in the glucose-related production of CO_2_. A simple way to test this hypothesis is to inhibit OXPHOS and to evaluate glucose oxidation capacity. OXPHOS blockade by rotenone (ROT, 1 μM) and/or antimycin (AA, 1 μM) prevented most of the B[a]P-increased glucose oxidation (Fig. 2B) although a slight, but significant, increase in glucose oxidation was still detected in B[a]P-treated cells under these conditions (Fig. 2B). In addition to enhanced glucose oxidation, we also found that pyruvate oxidation was enhanced following 24 h of exposure to B[a]P with a more marked increase at 48 hours (Fig. 2C). Altogether, these results suggest that increased oxidative capacities, following exposure to B[a]P, mainly occur through the tricarboxylic acid (TCA) cycle and might reflect alterations in electron transport chain complexes, since these capacities are strongly decreased upon OXPHOS inhibition.

### Implication of the mitochondrial complex II in the B[a]P-related TCA cycle alterations

Since the results above show that exposure to B[a]P results in both a decrease in oxygen consumption as well as increases in glucose and pyruvate oxidation, we hypothesized that B[a]P leads to a disconnection between the TCA cycle and OXPHOS. Inhibition of the respiratory chain complex II can trigger such an effect and lead to superoxide anion (O_2_^.−^) production^27^. We previously showed that O_2_^.−^ production was induced by B[a]P in F258 cells^28^. Therefore, we measured the two complex II-related activities, SQR [succinate:ubiquinone oxidoreductase] and SDH [succinate dehydrogenase]. B[a]P (50 nM, 48 h) significantly decreased SQR activity (~25% as compared to controls), without any effect on SDH activity (Fig. 2D). No change in complex I activity was detected (Supplementary Fig. S3B). Since acidification of the mitochondrial matrix can lead to similar effects on complex II^25^, we measured the matrix pH following treatment with B[a]P (50 nM, 48 h). It was decreased by about 1.5 pH unit (Supplementary Fig. S3C). Note that a mitochondrial hyperpolarization has previously been reported to be associated with matrix acidification in order to maintain the proton-motive force^29^. As expected^29^, the uncoupling agent FCCP induced an even more pronounced effect on matrix pH (Supplementary Fig. S3C). An increase in succinate concentration can result from complex II inhibition^27^,^30^,^31^. B[a]P (50 nM, 48 h) significantly enhanced the concentration of this metabolite while decreasing fumarate (Fig. 2E), further confirming the effects of B[a]P on complex II.

### Roles for AhR and NHE1 in B[a]P-induced metabolic reprogramming in F258 cells

**Figure 3:**
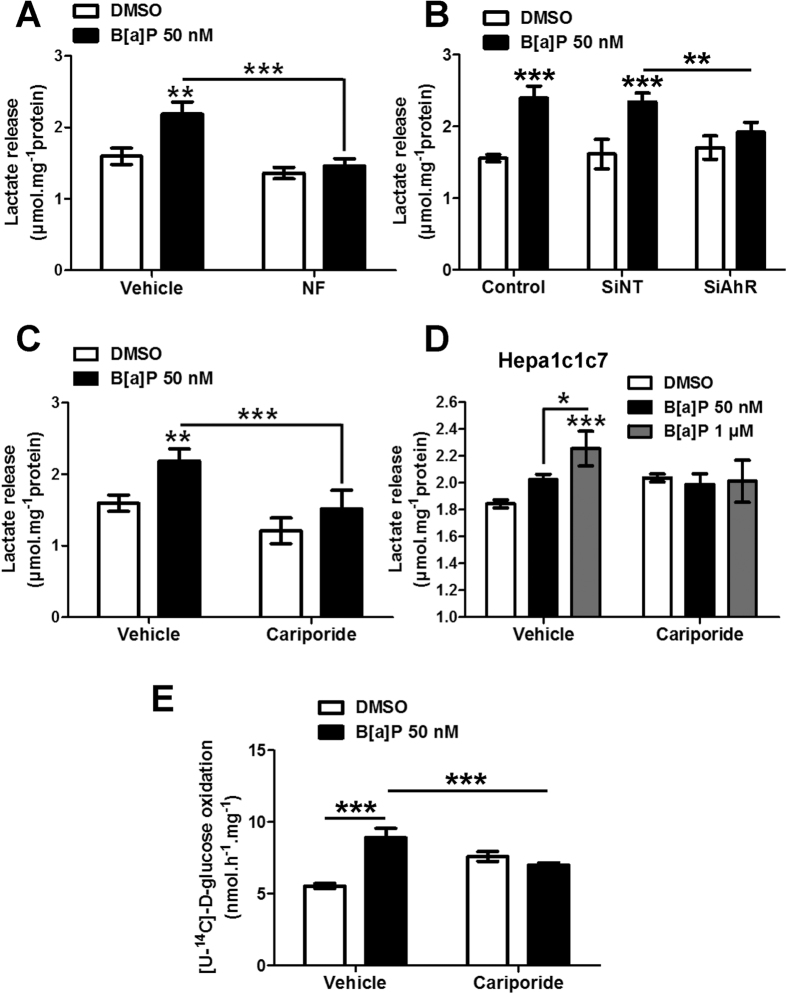
Metabolic reprogramming following B[a]P exposure involves both the AhR and NHE1 pathways. (**A**) To test the role of B[a]P metabolism and/or AhR in the glycolytic shift, F258 cells were pre-treated for 1 h with α-naphtoflavone (NF) (10 μM) prior to co-exposure with B[a]P (50 nM, 48 h). Following treatments, extracellular lactate release was monitored. (**B**) Effects of AhR inhibition using siRNA on the extracellular lactate release in B[a]P (50 nM, 48 h)-treated F258 cells. SiNT: non targeting SiRNA; SiAhR: AhR targeting siRNA. (**C,D**) Effects of NHE1 inhibition by cariporide (10 μM) on the B[a]P (50 nM or 1 μM, 48 h)-induced extracellular lactate release in F258 (**C**) or Hepa1c1c7 cells (**D**). Cells were pre-treated for 1 h with cariporide prior to co-exposure to B[a]P. (**E**) Effects of NHE1 inhibition by cariporide (10 μM) on the B[a]P (50 nM, 48 h)-elicited increase in glucose oxidation quantified by the release of radiolabelled CO_2_ in F258 cells. There were 3 to 4 independent experiments for all conditions. *p < 0.05, **p < 0.01; ***p < 0.001: DMSO *vs* B[a]P-treated cells, unless indicated by lines on graph.

We next tested whether CYP-related B[a]P metabolism was involved. Alpha-naphthoflavone (NF), a known inhibitor of CYP metabolism and of AhR, fully prevented the B[a]P (50 nM, 48 h)-induced lactate release (Fig. 3A). A possible role for the AhR receptor in the B[a]P-induced glycolytic shift was further tested. Silencing AhR through an siRNA prevented the B[a]P (50 nM, 48 h)-induced increase in lactate release (Fig. 3B), thus suggesting a role for the AhR in the B[a]P-induced metabolic deregulation. In addition, AhR activation by TCDD (10 nM, 48 h), a strong ligand and activator of this receptor, increased lactate release in F258 cells (Supplementary Fig. S4).

B[a]P metabolism activated an NHE1 pathway in F258 cells^15^,^23^. Since NHE1 is known to regulate both cell energy metabolism^32^,^33^ and complex II activities^25^, we evaluated its involvement in the B[a]P-induced glycolytic shift. To this end, we used cariporide (10 μM) to inhibit NHE1 activity. Co-exposure with cariporide eliminated the change in lactate release due to B[a]P (Fig. 3C) thus demonstrating that NHE1 is involved in this metabolic shift. Similar results were obtained in Hepa1c1c7 cells (Fig. 3D), in which B[a]P also activated NHE1^22^ and increased lactate release (Fig. 3D). In order to test whether NHE1 is involved also in the TCA cycle alterations, we measured glucose oxidation in the presence of cariporide. The increase in oxidation by B[a]P (50 nM, 48 h) was fully prevented by this inhibitor (Fig. 3E). Taken together, these results point to a role for the B[a]P-activated NHE1 pathway in metabolic reprogramming.

### Role for the B[a]P-induced glycolytic shift as a survival signal in F258 cells

**Figure 4:**
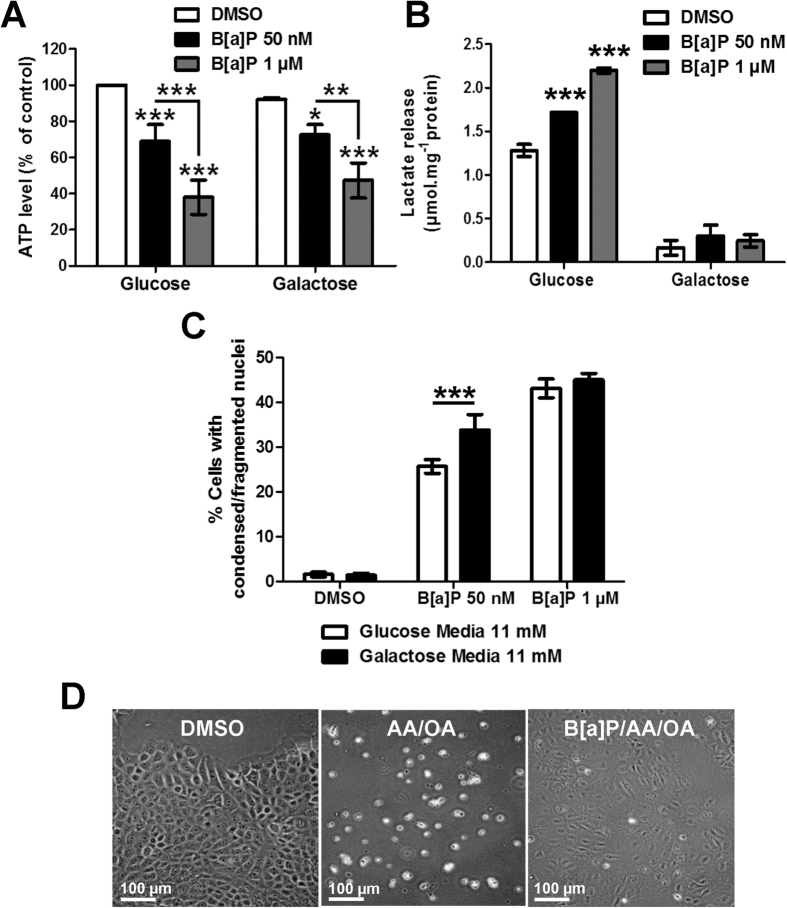
The glycolytic reprogramming occurring upon B[a]P exposure acts a survival signal in F258 cells. Cells were forced to rely on OXPHOS pathways by replacing glucose for galactose in the culture medium. Cells were cultured with galactose for at least 3 passages before starting the experiments. (**A**) ATP levels following treatment with B[a]P (50 nM or 1 μM; 72 h) using glucose and galactose media. (**B**) Extracellular lactate release using galactose or glucose media with or without B[a]P (50 nM or 1 μM, 48 h). (**C**) B[a]P (50 nM or 1 μM, 72 h)-induced cell death was analyzed by counting cells with fragmented or condensed chromatin following Hoechst 33342 staining, in galactose or glucose media. (**D**) Overall cell integrity was evaluated by optical microscopy following 48 hours of co-treatment with antimycin A (AA, 25 μM)/Oligomycin A (OA, 8 μM) to inhibit OXPHOS, with or without B[a]P (50 nM). There were ≥3 independent experiments for all conditions. *p < 0.05, ***p < 0.001: DMSO *vs* B[a]P-treated cells, unless indicated by lines on graph.

We next investigated the role for the glycolytic shift in the phenotypic responses to B[a]P. We evaluated the impact of B[a]P on cell death when glycolysis was prevented, upon forcing cells to rely on OXPHOS by substituting glucose by galactose in culture medium^34^,^35^. First, we found that, in the absence of any treatment, there was no significant change in the intracellular ATP level between glucose or galactose containing media. Further, the effects of B[a]P on level of ATP did not appear to be affected by the medium substrate (Fig. 4A). As expected, replacement of glucose with galactose blocked the B[a]P (50 nM or 1 μM, 48 h)-induced release of lactate (Fig. 4B). Replacement of glucose with galactose increased the 50 nM B[a]P (72 h)-induced cell death, as estimated by Hoechst staining (Fig. 4C). To further validate these results, we tested the effects of B[a]P (50 nM, 48 h) on F258 cells treated with antimycin A (AA, 25 μM) and oligomycin A (OA, 8 μM), which are inhibitors of respiratory chain complex III and ATP synthase, respectively. The concentrations for these experiments were chosen to induce drastic cell death under control conditions. Co-exposure with B[a]P favored survival of cells treated with AA/OA, that is, when OXPHOS was inhibited (Fig. 4D).

Altogether, these results point to glycolytic reprogramming as being responsible for a survival signal in B[a]P-treated cells.

### B[a]P affects F258 cell phenotype *via* an EMT-like process

**Figure 5:**
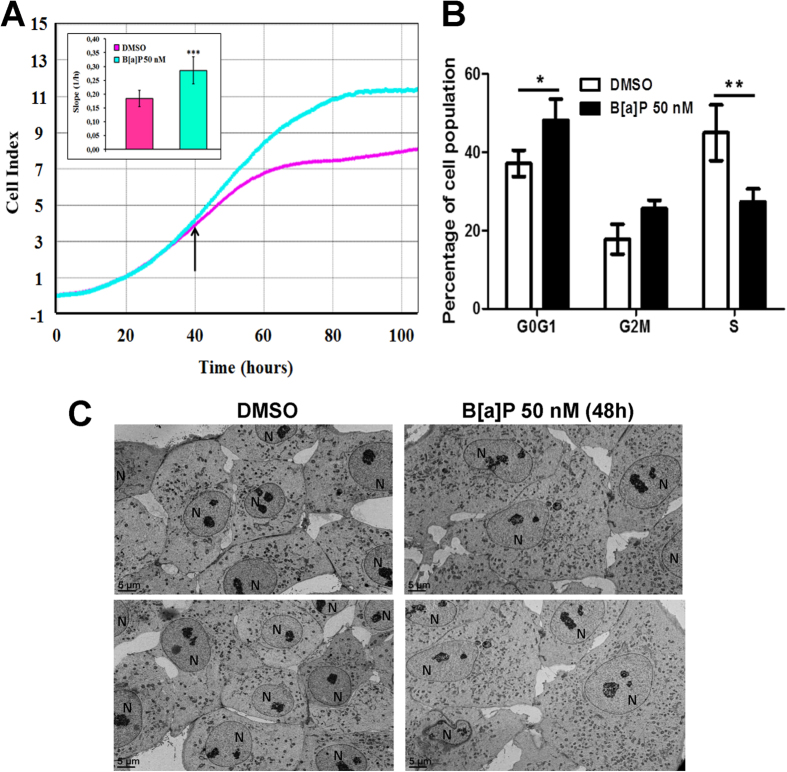
Effects of B[a]P on cell phenotype and cell cycle in F258 cells. Cells were treated or not with B[a]P 50 nM. (**A**) Cell attachment and spreading were analyzed by monitoring impedance of the cell monolayer with xCELLigence technology. The inset histogram plots the slope of cell index measured in presence or absence of B[a]P. There were 5 independent experiments. (**B**) Impact of B[a]P on cell cycle progression was evaluated by flow cytometry following IP staining. There were 3 independent experiments. *p < 0.05, **p < 0.01, ***p < 0.001. DMSO *vs* B[a]P-treated cells. (**C**) Analysis of B[a]P-induced cell spreading by observation of ultra-thin sections (90 nm) of each culture by transmission electron microscopy. N = nucleus. Number of independent experiments = 2.

Since glycolytic reprogramming is generally associated with cell survival as well as an epithelial-mesenchymal transition (EMT)^36^, we next performed measurements using xCELLigence technology to study real time changes in proliferation rates and/or in cell morphology. The increase in impedance, as shown by the cell index (CI) time course, was greater in the presence of B[a]P (50 nM) after 40 h, with a significant increase in the slope (about 75%; Fig. 5A, inset). Cell cycle analysis indicated that proliferation was hampered as shown by the decrease in S phase and cell accumulation in G0G1 phase (Fig. 5B). Taken together, these observations suggest that the B[a]P-induced CI increase might be the result of cell spreading. This conclusion is supported by the electron microscopic observations which show a higher surface area of cell cytoplasm in B[a]P-treated cells as compared to control cells (Fig. 5C).

**Figure 6:**
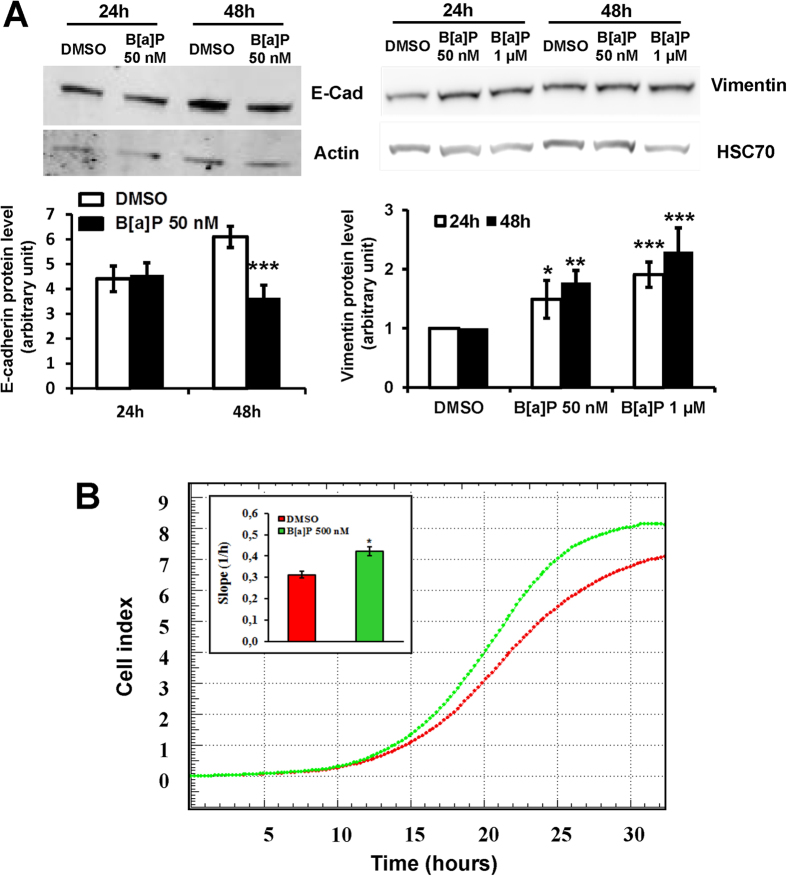
B[a]P triggers an EMT-like process and increases migratory potential in F258 cells. (**A**) E-cadherin and vimentin protein levels were analyzed by western blotting after 24 and 48 h of B[a]P treatment (50 nM or 1 μM). Histograms obtained from densitometric analysis of the western blots are shown below the blots. The level of the test protein is given relative to the level of HSC70. There were 3 independent experiments for all conditions. *p < 0.05, **p < 0.01, ***p < 0.001: DMSO *vs* B[a]P-treated cells. (**B**) Cell migration assays were monitored using xCELLigence technology on B[a]P (500 nM)-treated cells. The inset histogram shows the slope of cell index measured in the presence or absence of B[a]P. There were 3 independent experiments for all conditions. *p < 0.05: DMSO *vs* B[a]P-treated cells.

Finally, we looked for an EMT-like process upon B[a]P exposure. We first analyzed the same previously published transcriptome study (GSE40117^26^) as we did for the metabolic studies. The ssGSEA analysis revealed that the expression of an EMT-related gene set was globally up-regulated, notably the mesenchymal marker vimentin (Supplementary Fig. S5). In line with this, an increase in the level of vimentin protein was detected in F258 cells exposed to B[a]P (50 nM, 24 h), along with a downregulation of the epithelial marker E-cadherin (48 h) (Fig. 6A). Thus, B[a]P appeared to induce an EMT under our experimental conditions. Further support for this shift was obtained by using the xCELLigence technology with CIM plates to investigate the B[a]P impact on the F258 cell migration. Although we observed a trend toward an increase in migratory capacity of the cells after exposure to 50 nM B[a]P, a large variability in the response failed to give statistically significant results (data not shown). Therefore, 500 nM B[a]P was used (a condition without excessive toxicity) in order to increase the amplitude of the effect. A marked increase in the slope was detected upon B[a]P exposure (Fig. 6B), which suggests enhanced cell migration capacities.

Taken together, these results indicate that B[a]P triggers the appearance of a mesenchymal-like phenotype associated with an increased capacity for migration.

## Discussion

High intrinsic Δψm is a shared feature of many cancer types, and it is linked to their degrees of aggressiveness^5^,^6^. However, little is known about its pathophysiological origin. TCDD, a strong AhR activator, has been shown to trigger mitochondrial hyperpolarization^9^. In this context, chronic exposure to toxicants that target AhR could, thus, participate in cell transformation by sustainably increasing Δψm. In line with this, we previously demonstrated that B[a]P increased Δψm in F258 cells^20^,^21^. Similarly, hyperpolarization has been observed in low dose bisphenol A-treated HepG2 cells^37^. Since a link between high Δψm and metabolic reprogramming was proposed previously^8^, the present study was dedicated to analyze energy metabolism in F258 cells following exposure to low doses of B[a]P. Despite some literature which indicates a possible effect of B[a]P on glycolysis^38^,^39^ or OXPHOS^40^, metabolic reprogramming and its role in the cell responses induced by environmental carcinogens remain to be investigated for B[a]P as for many other environmental contaminants^41^. Here, we show for the first time that exposure to a low concentration of B[a]P can lead to a Warburg effect in hepatic cells (three different cell lines) which is characterized by a fall in respiratory rate and an enhancement of lactate production (see Supplementary Fig. S6 for a graphic summary of the present results). It is worth emphasizing that DMBA, another PAH carcinogen, and TCDD also were shown here to enhance lactate release. Therefore, such metabolic reprogramming might provide new insights into the carcinogenic process elicited by aromatic hydrocarbons, since the Warburg effect is a core hallmark of cancer cells^1^.

B[a]P also altered the TCA cycle in F258 cells by producing an increase in both glucose and pyruvate oxidation capacities. The fact that oxygen consumption was markedly decreased, suggested a disconnection between the TCA cycle and the respiratory chain complexes. Such a disconnection is supported by our data which show a decrease in the complex II SQR activity. A recent proteomics analysis of Hepa1c1c7 cells reported effects of B[a]P on both glycolysis and the TCA cycle^42^. However, these findings were obtained with a relatively high concentration of B[a]P (5 μM), and they were based only on levels of protein expression, unlike our study which deals with metabolite levels and enzyme activities. Furthermore, in contrast to our results, that study described a decrease in glycolysis and an increase in OXPHOS following a 24 h-treatment. These differences might stem from the different cell phenotypes used (Hepa1c1c7 cells are hepatoma cells; F258 cells are spontaneously transformed cells), from differences in the metabolism of B[a]P and/or sensitivity towards this carcinogen^22^, or from the concentrations employed. Regarding the latter possibility, it is noteworthy that we observed enhanced lactate production in Hepa1c1c7 cells following exposure to B[a]P (48 h, 50 nM or 1 μM). Also, our ssGSEA analysis of previously published data on B[a]P (2 μM, 72 h)-treated HepG2 cells (Supplementary Fig. S1) are in line with the results presented here. Regarding the origin of the complex II dysfunction, mitochondrial matrix acidification, which was observed here, might be involved, as previously suggested^25^.

B[a]P metabolism activates NHE1 in F258 cells, which leads to an intracellular alkalinization^15^,^23^. NHE1 activation has been implicated in cell malignant transformation, notably by regulating the glycolytic shift^19^. Similarly, this activation is required for the glycolytic shift in F258 and Hepa1c1c7 cells, in which B[a]P induces an alkalinization^22^,^23^. This is in line with the observation that cytosolic alkalinization stimulates glucose utilization in cultured hepatocytes^33^. This role for NHE1 in the B[a]P-induced glycolytic shift might stem from the known pH-dependent regulation of glycolytic enzyme activities like phosphofructokinase^43^. It is worth emphasizing that the activities of hexokinase, phosphofructokinase, pyruvate kinase and lactate dehydrogenase are increased in the lungs of B[a]P-treated mice^38^. In the present study, NHE1 appeared to control the B[a]P-increased glucose oxidation. A role for active NHE1 in upregulating glucose oxidation and the TCA cycle rate thereby participating in the prevention of ischemia-reperfusion injury in the hearts of NHE1 transgenic mice has been reported previously^44^. Under our conditions, an effect of pH on enzyme activities involved in TCA cycle could be proposed. Besides NHE1, our results also demonstrate a role for AhR in the glycolytic shift. Since this receptor is responsible for membrane remodeling and, hence, NHE1 activation induced by B[a]P^14^,^45^, an effect of AhR on the glycolytic shift *via* an effect on NHE1 activity might occur (Supplementary Fig. S6). Other possibilities might involve the previously described role of AhR on ΔΨm^9^, or on glucose metabolism enzyme expression^46^.

A strong toxicity of OXPHOS inhibitors was observed in the absence of B[a]P (Fig. 4D). This suggests that, under normal conditions, F258 cell mitochondria are fully functional. Treatment with B[a]P (50 nM), interestingly, allowed cells to survive OXPHOS inhibition, which suggests a cellular adaptation and a metabolic plasticity due to B[a]P. This flexibility could confer to B[a]P-exposed cells a better energetic phenotype that would allow survival under deleterious conditions. As demonstrated in this work, B[a]P both increased glycolysis and partly stimulated the TCA cycle. The B[a]P-induced Warburg effect was found to support cell survival since F258 cells which were forced to rely on OXPHOS (by a replacement of glucose by galactose) became more sensitive to the toxic action of B[a]P despite similar intracellular ATP concentrations. Interestingly, the protection afforded by glycolysis was observed only with low doses of B[a]P which suggests that, for higher concentrations, pro-survival signals might be overwhelmed by death signals, despite higher lactate levels. How glycolysis initiates a survival pathway under our conditions remains to be elucidated. One clue might come from the PPP. Indeed, even though this pathway seems to be only weakly induced by B[a]P, the induction might suffice to increase intracellular levels of glutathione or of reducing equivalents such as NADPH^47^, thereby limiting the B[a]P-induced oxidative stress^48^. Another possibility might rely on complex II dysfunction. Indeed, the inhibition of complex II activity is known to increase the succinate level. This can then favor cell survival notably by activating the hypoxia-inducible factor-1α (HIF-1α) transcription factor^49^. Based upon the fact that the level of succinate increased upon exposure to B[a]P, one might then infer an activation of HIF-1α which would result in the stimulation of glycolysis^50^. Complex II dysfunction also has been related to mitochondrial ROS production, with either “deleterious ROS” or “signaling ROS”, which support either cell death or survival, respectively^51^. Since mitochondrial O_2_^.−^ production is increased in exposed F258 cells^28^, one might infer a role for O_2_^.−^ in triggering survival pathways. However, it is noteworthy that the use of anti-oxidant molecules prevents B[a]P-induced cell death, thus pointing to “deleterious” rather than “signaling” ROS^15^,^23^,^28^. In this context, B[a]P-induced complex II dysfunction might play an ambivalent role, in both survival and cell death. With respect to cell phenotype, recent work has shown that chronic exposure to low concentrations of B[a]P does not alter hepatocarcinoma cell growth but promotes cell migration and invasion both *in vitro* and *in vivo*^12^. The fact that we observe both a survival supported by the glycolytic shift and an EMT upon exposure to B[a]P might, thus, indicate a link between these two phenomena, as already reported^52^. This link will have to be explored further.

In conclusion, we have shown here that exposure to B[a]P can trigger a Warburg-like metabolic shift, that supports hepatic cell survival, and, ultimately, leads to modifications of cell phenotype and migration. We, therefore, assume that such effects also might participate in the carcinogenic potential of B[a]P.

## Additional Information

**How to cite this article**: Hardonnière, K. *et al*. The environmental carcinogen benzo[a]pyrene induces a Warburg-like metabolic reprogramming dependent on NHE1 and associated with cell survival. *Sci. Rep.* **6**, 30776; doi: 10.1038/srep30776 (2016).

## Supplementary Material

Supplementary Information
