# Supplementary material for: The environmental carcinogen benzo[a]pyrene induces a Warburg-like metabolic reprogramming dependent on NHE1 and associated with cell survival
Source: Sci Rep. 2016 Aug 4;6:30776. doi: 10.1038/srep30776 (PMC4973274; doi:10.1038/srep30776)
Supplement: Supplementary Information [file srep30776-s1.pdf]

SUPPLEMENTARY INFORMATION

The environmental carcinogen benzo[a]pyrene induces a Warburg-like metabolic reprogramming dependent on NHE1 and associated with cell survival

Kévin Hardonnière[1,2], Elise Saunier[3], Anthony Lemarié[4], Morgane Fernier[1,2], Isabelle Gallais[1,2], Cécile Héliès-Toussaint[5], Baharia Mograbi[6], Samantha Antonio[3], Paule Bénit[7,8], Pierre Rustin[7,8], Maxime Janin[3,9], Florence Habarou[3,9], Chris Ottolenghi[3,9], Marie-Thérèse Lavault[2], Chantal Benelli[3], Odile Sergent[1,2], Laurence Huc[5$], Sylvie Bortoli[3$], Dominique Lagadic-Gossmann[1,2$*]

SUPPLEMENTARY METHODS

In silico tests for B[a]P-affected metabolic signatures

The single sample Gene Set Enrichment Analysis (ssGSEA) method was applied on the published microarray dataset of B[a]P-exposed HepG2 GSE40117 (Doktorova et al., 2013), to determine the B[a]P-affected metabolic signatures. Data were downloaded from the InSilico DB Genomic Datasets Hub (https://insilicodb.com/; Coletta et al., 2012), and analyzed by applying the ssGSEA method available in the GenePattern software. (http://genepattern.broadinstitute.org/). The ssGSEA method provides a representation of the gene expression data by assigning to each individual sample an Enrichment Score (ES) with respect to each gene set. ssGSEA. Heatmap visualization of the ssGSEA was then performed using GENE-E (http://www.broadinstitute.org/).

Glucose uptake assay

Glucose uptake was measured according to Kim et al (Kim et al., 2010) with some modifications. Briefly, after 48 hours of B[a]P treatment, cells were washed twice with serum-free, glucose-free William's medium supplemented with 0.1% BSA and pre-incubated with this medium for 3 hours at 37°C. After a starvation period, cells were washed twice with Krebs-Ringer-Bicarbonate buffer (KRB), and incubated further for 30 minutes at 37°C with 100 nM insulin (or not, for the negative control). To initiate glucose uptake, 2-deoxy-[1-^{3}H]-glucose (1µCi/mL), diluted in 0.1mM 2-deoxyglucose solution, was added to each well and further incubated for 10 minutes at 37°C. After incubation, cells were washed twice with ice-cold KRB and solubilized with 0.1N NaOH. Half of the content of each well was transferred into scintillation vials, and 10 mL of scintillation cocktail, Ultima Gold LLT, were added. The radioactivity incorporated into cells was measured using a liquid scintillation counter (Hewlett Packard, USA). The protein content was assayed for each point on the remaining half with the Pierce, bicinchoninic acid enzymatic kit (Pierce, France) after cell lysis in 0.1N NaOH. The results were expressed as the radioactivity incorporated related to the protein content.

Flow cytometry analysis of cell cycle

After a 48h-B[a]P treatment, cells were harvested and washed with phosphate-buffer saline (PBS). Cell nuclei were stained with propidium iodide using the Cycle Test™ PLUS DNA Reagent Kit (Becton Dickinson, San Jose, CA). DNA content of 20 000 cells/analysis was then monitored with a FACSCalibur flow cytometer (Becton Dickinson). Analysis of the cell cycle parameters was performed using the Modfit software (Becton Dickinson).

Analysis of cell phenotype using Transmission Electron Microscopy (TEM)

After drug exposure, cells were rinsed with 0.15 M Na cacodylate buffer and fixed by drop-wise addition of glutaraldehyde (2.5%) for 1 h. After fixation, the specimens were rinsed several times with 0.15 M Na cacodylate buffer and postfixed with 1.5% osmium tetroxide for 1 h. After further rinsing with cacodylate buffer, the samples were dehydrated with a series of ethanol solutions from 70 to 100%. The specimens were infiltrated in a mixture of acetone–Eponate (50/50) for 3 h and then in pure Eponate for 16 h. Finally, the specimens were embedded in DMP30–Eponate for 24 h at 60 °C. Sections (0.5 µm) were cut on a Leica UC7 microtome and stained with toluidine blue. Ultrathin sections (90 nm) were obtained, collected onto copper grids, and counterstained with 4% uranyl acetate and then with lead citrate. Examination was performed with a JEOL 1400 transmission electron microscope operated at 120 kV.

Western blotting

After treatment, cells were harvested and lysed for 20 min on ice in RIPA buffer supplemented with 1 mM phenylmethylsulfonyl fluoride, 0.5 mM dithiothreitol, 1 mM orthovanadate, and a cocktail of protein inhibitors (Roche). Cells were then centrifuged at 13,000g for 15 min at 4 °C.

Thirty µg of whole-cell lysates were heated for 5 min at 100 °C, loaded in a 4% stacking gel, and then separated by 10% sodium dodecyl sulfate–polymerase gel electrophoresis (SDS–PAGE). Gels were electroblotted overnight onto nitrocellulose membranes (Millipore). After membrane blocking with a Tris-buffered saline (TBS) solution supplemented with 5% bovine serum albumin, membranes were hybridized with primary antibodies against E-cadherin or vimentin overnight at 4 °C, and next incubated with appropriate horseradish peroxidase-conjugated secondary antibodies for 1 h. For protein loading evaluation, primary antibodies against HSC70 or β-actin were used. Immunolabeled proteins were then visualized by chemiluminescence using the LAS-3000 analyzer (Fujifilm). Image processing was performed using Multi Gauge software (Fujifilm).

Evaluation of mitochondrial pH

The pH_m was monitored using two mitochondrial targeted plasmids (Aequotech, Ferrara, Italy), both coding for cytochrome c oxydase subunit 8A mRNA: the mt-HA-eGFP pH-sensitive Green fluorescent protein (λex.=488 nm, λem.=509 nm), and the mt-dsRed pH-insensitive red fluorescent protein (λex.=530 nm, λem.=583) used as a transfection rate control. Production of these two plasmids was performed using the PureLink HiPure Plasmid Filter Maxiprep Kit (Life Technologies). F258 cells were co-transfected for 24h with both plasmids using X-tremeGene HP DNA Transfection Reagent (Roche, Meylan, France). After 24 h, cells were treated with B[a]P for 24 or 48h. Cells were then collected, re-suspended in Cell Suspension Buffer (see Huc et al., 2004, for CSB composition), and analysed by flow cytometry. Mean Fluorescence Intensity (MFI) was determined on 40 000 cells using a FACSCalibur (BD Bioscience). Fluorescence intensity was analyzed using the standard laser 488 nm laser filter configuration with FL1-H and FL3-H channels for monitoring mt-HA-eGFP and for DsRed respectively. A standard curve was generated *in situ* on control cells exposed to calibration buffers containing ionophores nigericin and monensin, allowing the conversion of the MFI ratio 530/640 into pH value. FCCP, a mitochondrial protonophore, was used as control for pHm acidification.

Evaluation of complex I activity

Complex I activity was measured on frozen cells as described (Bénit et al., 2008). In brief, cells were mixed with 1 ml extraction buffer containing 0.25 M sucrose 20 mM Tris–HCl, 40 mM KCl, 2 mM EGTA, 1 mg/ml BSA, pH 7.2 (medium A). Digitonin (0.01% final) and Percoll (10%) were added for 5 min on ice. Cells were subsequently spun down at 2,500 g for 5 min and washed two times with 1 ml of medium A. Permeabilized cell pellet was subsequently used for enzyme measurement.

References

Bénit P., Slama A., Rustin P. 2008. Decylubiquinol impedes mitochondrial respiratory chain complex I activity. *Mol. Cell. Biochem.,* 314, 45-50.

Coletta A., Molter C., Duqué R., Steenhoff D., Taminau J., de Schaetzen V., Meganck S., Lazar C., Venet D., Detours V., Nowé A., Bersini H., Weiss Solís D.Y. 2012. InSilico DB genomic datasets hub: an efficient starting point for analyzing genome-wide studies in GenePattern, Integrative Genomics Viewer, and R/Bioconductor. *Genome Biol.,* 13, R104.

Doktorova T.Y., Yildirimman R., Vinken M., Vilardell M., Vanhaecke T., Gmuender H., Bort R., Brolen G., Holmgren G., Li R., Chesne C., van Delft J., Kleinjans J., Castell J., Bjorquist P., Herwig R., Rogiers V. 2013. Transcriptomic responses generated by hepatocarcinogens in a battery of liver-based in vitro models. *Carcinogenesis*., 34, 1393-1402.

Kim J., Park J., Kim E.-K., Lee J., Lee S., Jung J., You G., Park S., Suh P.-G., Kim H. 2010. Curcumin stimulates glucose uptake through AMPK–p38 MAPK pathways in L6 myotube cells. *J. Cell. Physiol.*, 223, 771–778.

Supplementary Figure S1

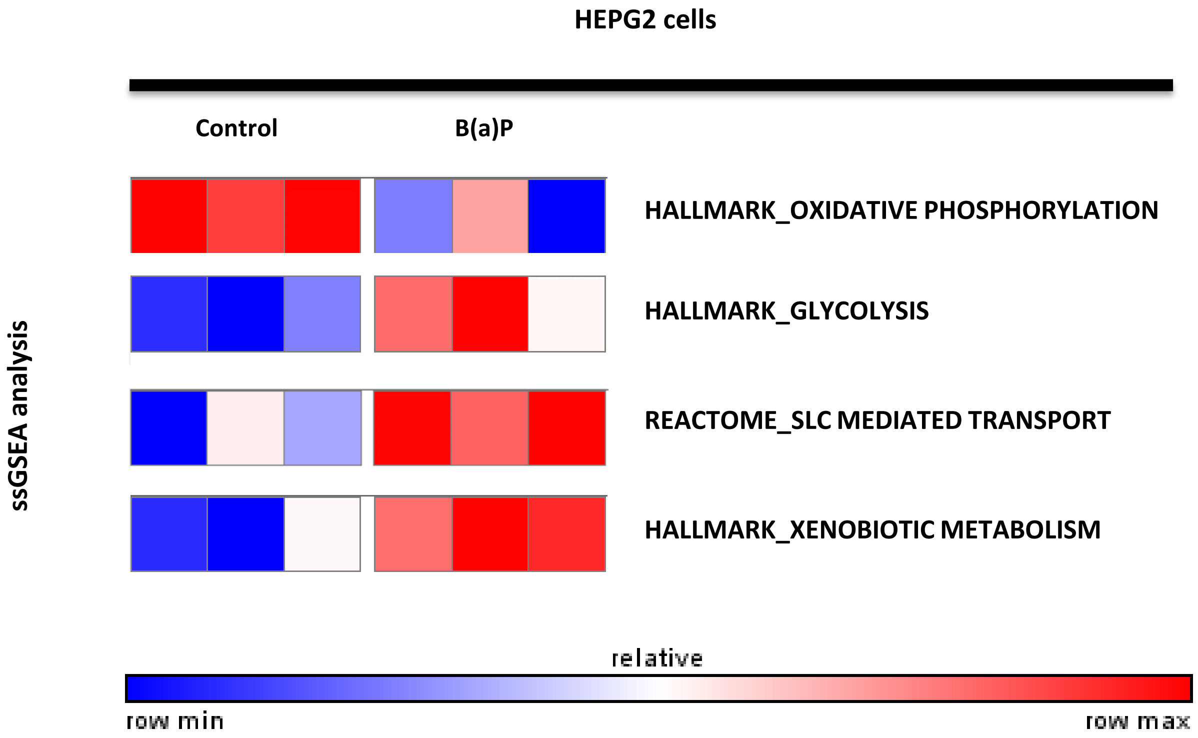

ssGSEA analysis of the metabolic shifts induced by B[a]P in HepG2 cell line. Heatmap of ssGSEA score of the published microarray dataset GSE40117 (Doktorova et al., 2013) showing the metabolic shift signatures affected by B[a]P (2 µM, 72h) exposure.

Supplementary Figure S2

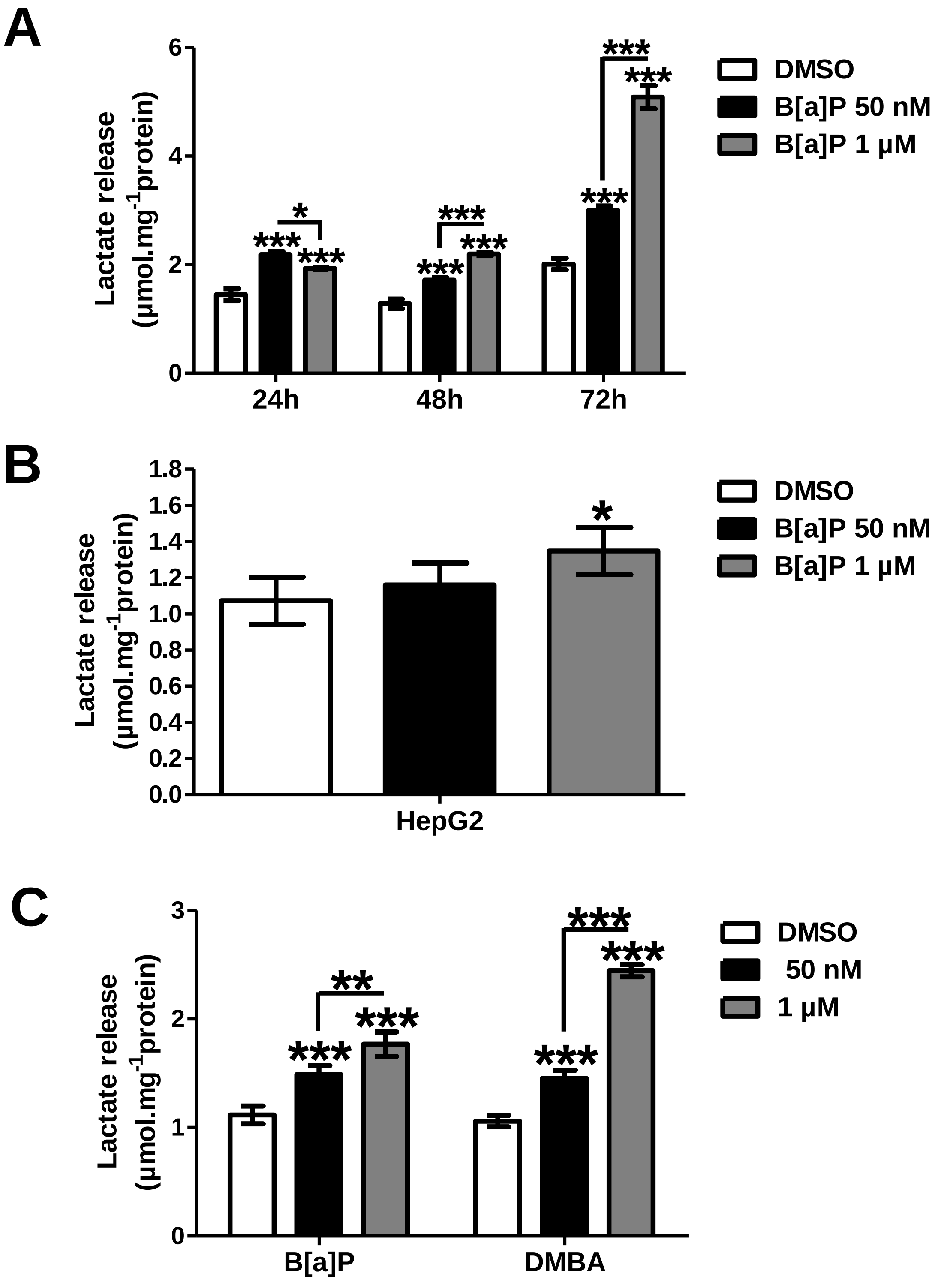

(A) Kinetic effect of B[a]P (50 nM or 1 µM) on the extracellular lactate release analyzed in F258 cells. (B) Extracellular lactate release in response to B[a]P (50 nM or 1 µM, 48h) was measured in HepG2 cells. (C) Effect of another well-known carcinogenic PAH, dimethylbenzanthracene (DMBA; 50 nM, 48h), on the extracellular lactate release analyzed in F258 cells. Number of independent experiments = 3 for all conditions. *p<0.05, **p<0.01,***p<0.001: DMSO *vs* B[a]P-treated cells, unless indicated by lines on graph.

Supplementary Figure S3

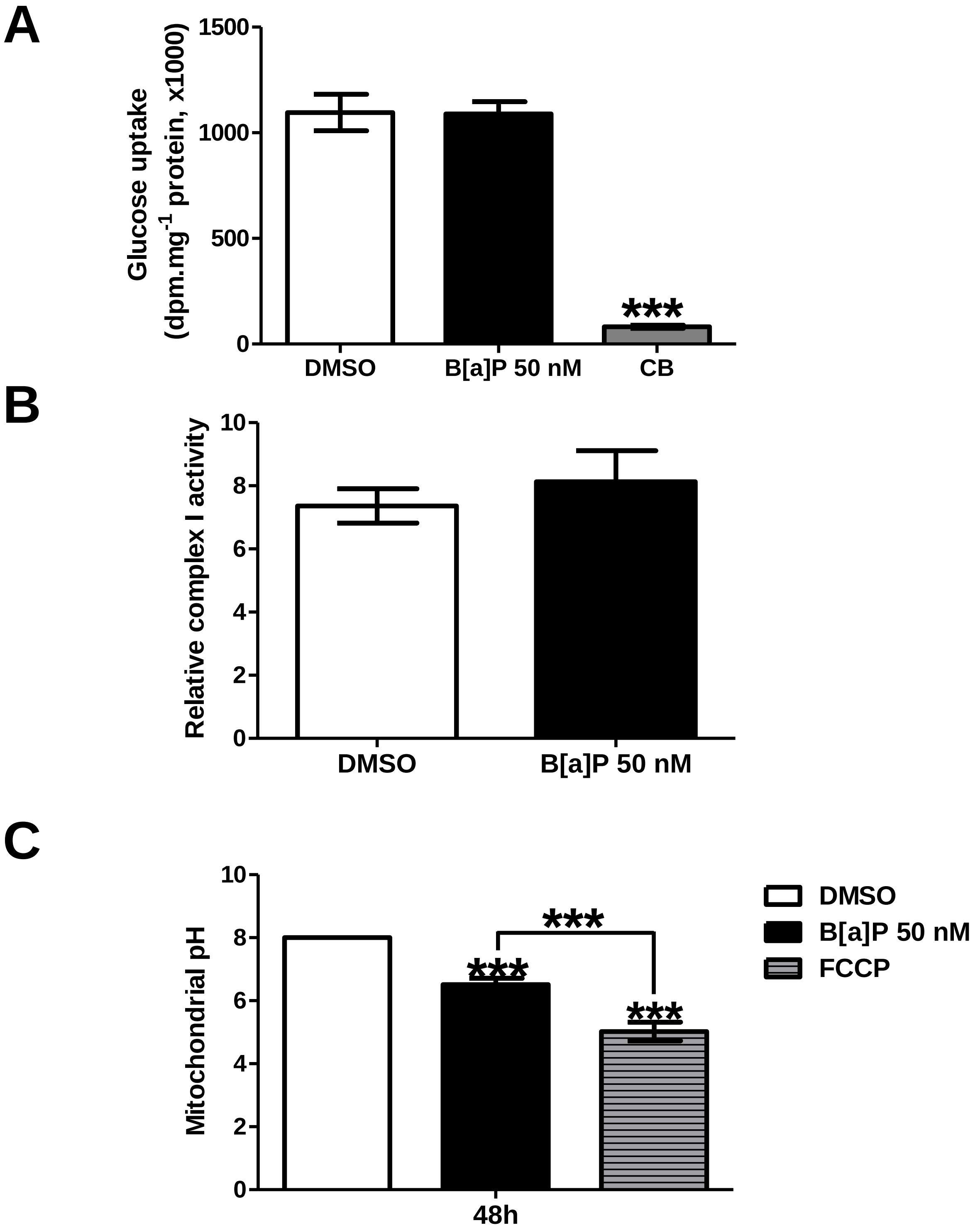

(A) Impact of B[a]P (50 nM, 48h) on glucose uptake in F258 cells. Glucose uptake was measured as described in Supplementary Material and Methods. Cytochalasin B (CB) was used to inhibit glucose uptake. (B) Effects of B[a]P (50 nM, 48h) exposure on complex I activity in F258 cells. (C) Impact of B[a]P (50 nM, 48h) on mitochondrial matrix pH. This pH was ratiometrically monitored by flow cytometry using two mitochondrial plasmids coding for fluorescent subunit 8A of the cytochrome c oxidase. FCCP was used as a positive control of mitochondrial matrix acidification. Number of independent experiments = 3 for all conditions. ***p<0.001: DMSO *vs* B[a]P-treated cells, unless indicated by lines on graph.

Supplementary Figure S4

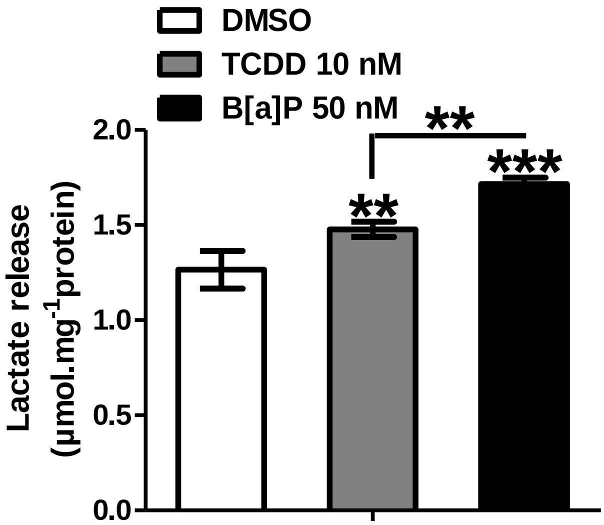

Effects of AhR activation by TCDD (10 nM, 48h) on the extracellular lactate release in F258 cells; the effects of B[a]P (50 nM, 48h) were plotted for comparison. Number of independent experiments = 3 for all conditions. **$p<0.01$,***$p<0.001$: DMSO *vs* B[a]P-treated cells, unless indicated by lines on graph.

Supplementary Figure S5

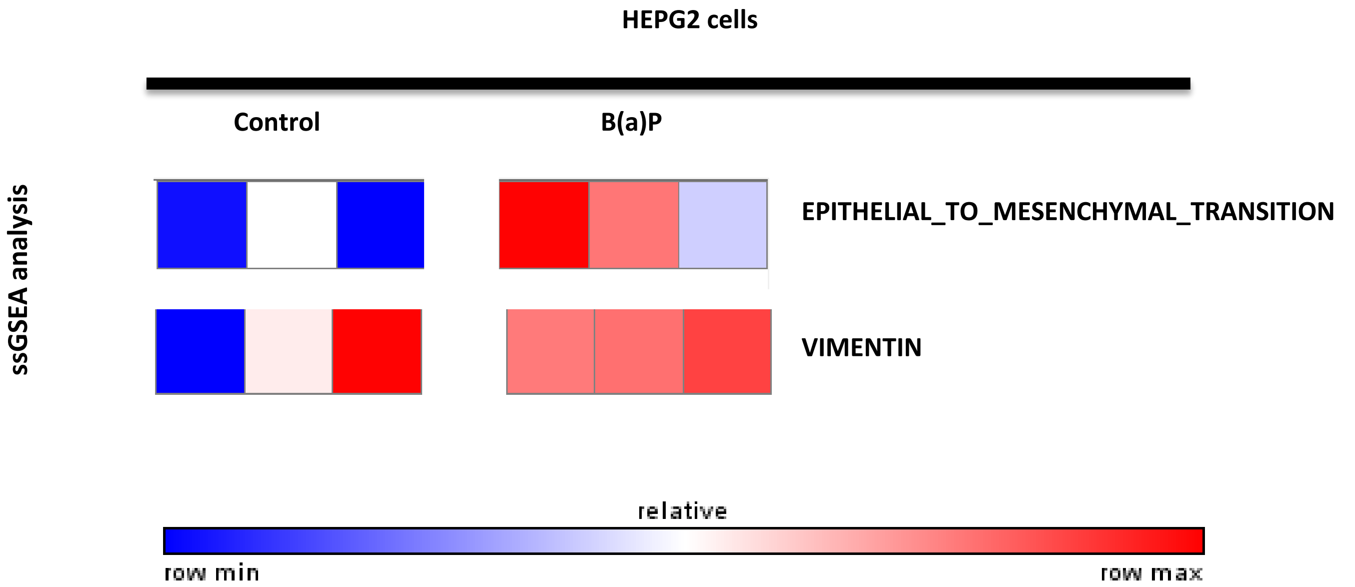

ssGSEA analysis of the epithelial to mesenchymal transition induced by B[a]P in the human hepatocarcinoma HepG2 cell line. Heatmap of published microarray dataset GSE40117 (Doktorova et al., 2013) showing the EMT shift signatures (ssGSEA score) and the mRNA expression of the EMT marker vimentin affected by B[a]P (2 µM, 72h) exposure.

Supplementary Figure S6

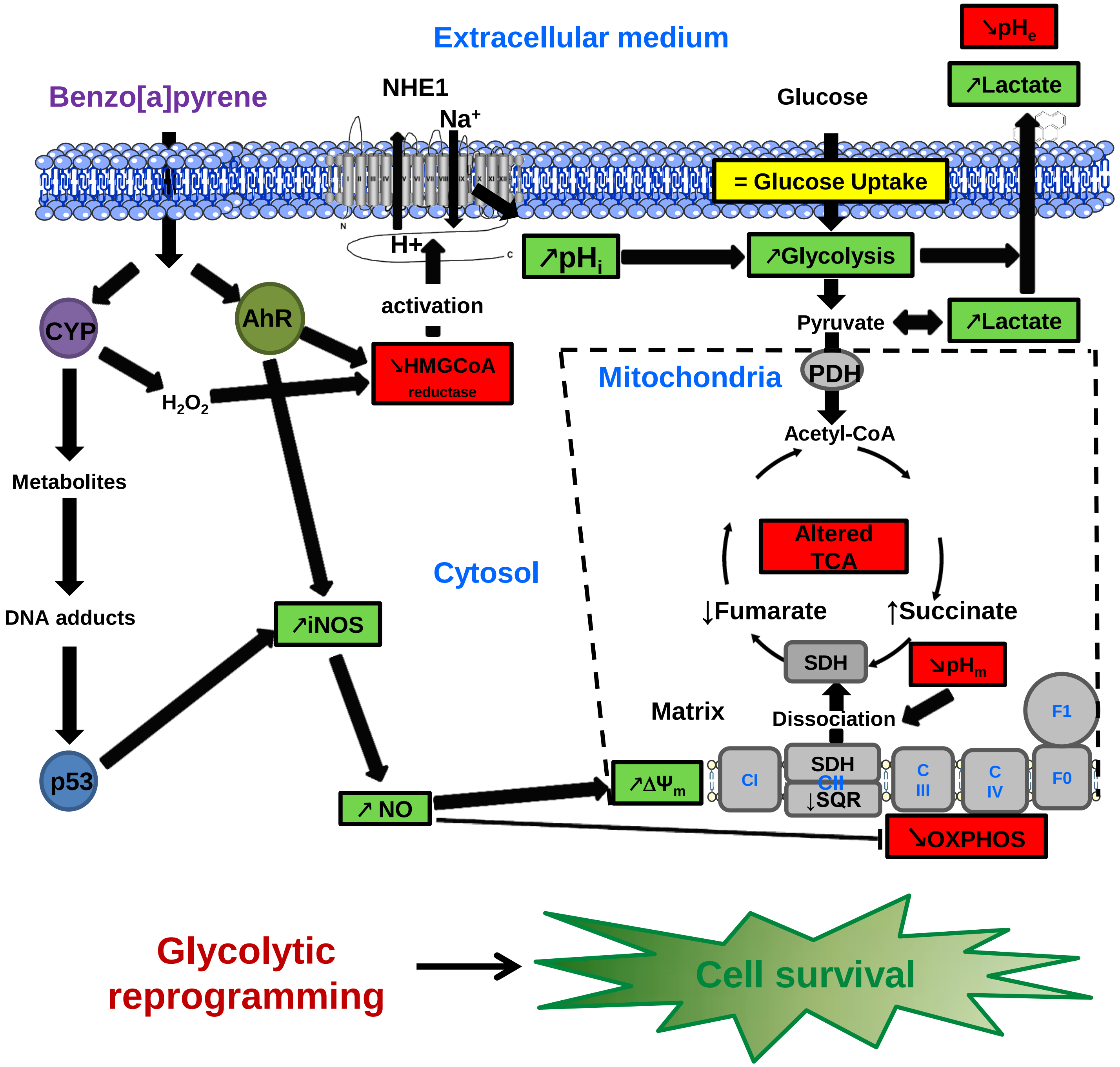

Schematic diagram illustrating the impact of B[a]P on cell metabolism in F258 rat hepatic epithelial cell line. Upon B[a]P exposure, a shift from OXPHOS to glycolysis is induced. Alterations in OXPHOS rely upon a mitochondrial complex II dysfunction that might result from dissociation triggered by acidification of matrix pH (pHm). Such a dissociation is associated with a decrease of fumarate and a parallel increase in succinate concentration. Stimulation of glycolysis which results in lactate production and extracellular pH (pHe) acidification, is dependent on NHE1 activation as reflected by an increased intracellular pH (pHi). A role for AhR has also been detected with regard to glycolysis that might go through the NHE1 activation resulting from AhR and H_2O_2-dependent membrane remodeling (Tekpli et al., 2010, 2012). Finally, a nitric oxide (NO) production also triggers mitochondrial dysfunction leading to membrane hyperpolarization (Hardonnière et al., 2015). In total, the B[a]P-induced glycolytic shift is involved in cell survival. *AhR: Aryl hydrocarbon receptor; CYP: Cytochrome P450; CI, CII, CIII, CIV: mitochondrial complexes I to IV of the respiratory chain; iNOS: inducible nitric oxide synthase; PDH: pyruvate dehydrogenase; SDH: succinate dehydrogenase; SQR: succinate:ubiquinone oxidoreductase; TCA: tricarboxylic acid cycle; $\Delta\psi m$: mitochondrial membrane potential.*
